# ConfluentFUCCI for fully-automated analysis of cell-cycle progression in a highly dense collective of migrating cells

**DOI:** 10.1371/journal.pone.0305491

**Published:** 2024-06-26

**Authors:** Leo Goldstien, Yael Lavi, Lior Atia

**Affiliations:** Department of Mechanical Engineering, Ben-Gurion University of the Negev, Beer-Sheva, Israel; University of Bayreuth, GERMANY

## Abstract

Understanding mechanisms underlying various physiological and pathological processes often requires accurate and fully automated analysis of dense cell populations that collectively migrate. In such multicellular systems, there is a rising interest in the relations between biophysical and cell cycle progression aspects. A seminal tool that led to a leap in real-time study of cell cycle is the fluorescent ubiquitination-based cell cycle indicator (FUCCI). Here, we introduce ConfluentFUCCI, an open-source graphical user interface-based framework that is designed, unlike previous tools, for fully automated analysis of cell cycle progression, cellular dynamics, and cellular morphology, in highly dense migrating cell collectives. We integrated into ConfluentFUCCI’s pipeline state-of-the-art tools such as Cellpose, TrackMate, and Napari, some of which incorporate deep learning, and we wrap the entire tool into an isolated computational environment termed container. This provides an easy installation and workflow that is independent of any specific operation system. ConfluentFUCCI offers accurate nuclear segmentation and tracking using FUCCI tags, enabling comprehensive investigation of cell cycle progression at both the tissue and single-cell levels. We compare ConfluentFUCCI to the most recent relevant tool, showcasing its accuracy and efficiency in handling large datasets. Furthermore, we demonstrate the ability of ConfluentFUCCI to monitor cell cycle transitions, dynamics, and morphology within densely packed epithelial cell populations, enabling insights into mechanotransductive regulation of cell cycle progression. The presented tool provides a robust approach for investigating cell cycle-related phenomena in complex biological systems, offering potential applications in cancer research and other fields.

## Introduction

**Fig 1:**
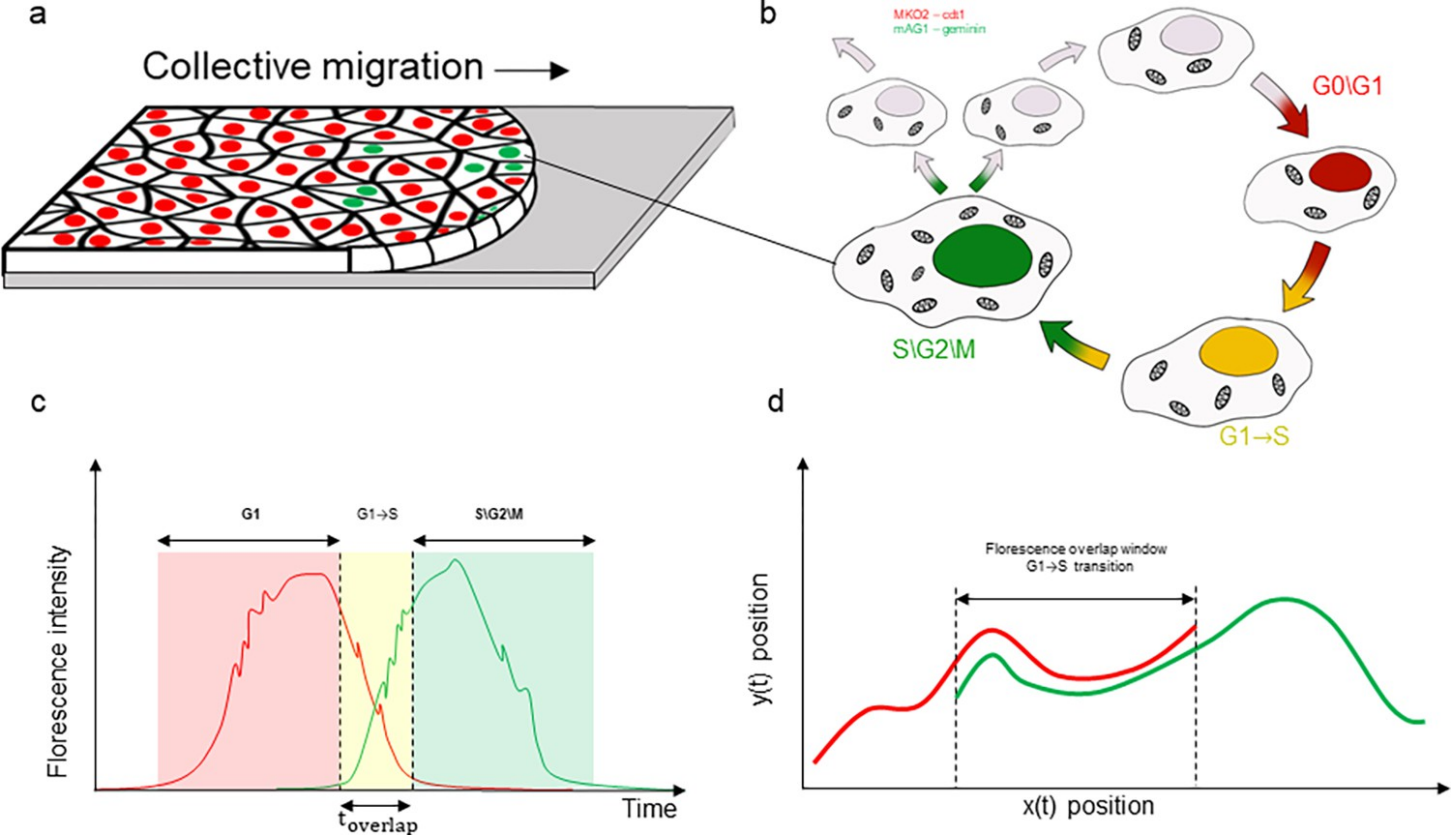
FUCCI monitoring during collective cellular migration. **a**, collective migration of confluent epithelial layer with cells tagged for FUCCI which, in **b** and **c** display different fluorescent colors as each cell progresses through the cell cycle. Newly formed cells are colorless. As G0\G1 progresses, the amount of cdt1 in the nucleus increase, and with it, the red fluorophore. When transitioning from G1 to S: 1) cdt1 concentration decreases, and with it, the red fluorophore gradually drops; 2) geminin concentration increases as shown by the rise in the green signal; 3) both fluorophores co-fluoresces and the cell appears yellow. When S progresses the red fluorophore disappears, while the appearance of green fluorophore continues to increase. Upon division, the green color is lost, with the disappearance of geminin. **d**. As each cell, among thousands in the layer, migrate, we track its position within each of the fluorescent channels, and while the two tracks are not perfectly aligned, our system automatically pairs red and green tracks that belongs to the same cells and consolidate them into one continues track with three phases: Red, Yellow, Green.

The cell cycle is a highly regulated process that governs the growth and division of cells. It consists of the sequential stages G1→S→G2→M (Fig 1) which include: synthesizing biomolecules for cell growth and function (G1); DNA replication (S); rapid cell growth and regulation of DNA damage (G2); chromosome segregation and cytokinesis (M), which together result in the formation of two daughter cells. In addition to the four stages mentioned, there is a quiescent stage in which the cell is metabolically active but not actively dividing (G0). Disruptions in the cell cycle can lead to various pathological disorders, including cardiovascular disease, infection, inflammation, and cancer [1]. The progression of the cell cycle is tightly controlled by signaling pathways that are intracellular, integrated with mechanical cues that are intercellular. Such mechanical cues stem from the extracellular matrix and neighboring cells [2]. These mechanotransductive processes are likely to be highly pronounced when neighboring cells in a dense tissue apply substantial forces between one another during collective cellular migration [3]. In the context of collective migration, specifically of epithelial cells, the regulation of cell cycle progression is crucial for ensuring the coordinated movement of cells. Therefore, understanding the relationship between cell cycle progression and collective cellular migration is essential for elucidating the mechanisms that govern various pathophysiologies and developing new therapeutic strategies to treat diseases, involving abnormal cell migration.

Several studies have made significant advancements in the study of cell cycle progression during collective migration using the fluorescence ubiquitination-based cell cycle indicator (FUCCI) [4, 5]. FUCCI is a seminal tool that led to a phenomenal leap in real-time study of cell cycle progression under various organisms and protocols [6, 7].

The FUCCI sensor utilizes two distinct fluorescent proteins conjugated to specific regulating proteins at different cell cycle stages: MKO2 to cdt1 (G0/G1) and mAG1 to geminin (S/G2/M). As cdt1 decreases and geminin increases, the overlay of the two channels displays the nuclei in yellow (G1 to S transition), which marks cell cycle progression (Fig 1). However, the inherent design of the FUCCI sensor, which indicates decreases or increases in cdt1 and geminin, makes both fluorescent images very heterogeneous. This creates significant computational challenges for image analysis in the context of collective migration. First, typical time-lapse imaging of collective migration experiments generates extremely large data sets per experiment and thus require a fully automated analysis. Second, the FUCCI fluorescent signals exhibits a wide dynamic range both in space and time. This feature of the system makes selecting analysis parameters (such as thresholds, SNR bounds, etc.) challenging and error prone. Finally, the extreme variability and heterogeneity makes marking the transition from G1 to S tied to a specific analyst opinion, and thus non-robust.

For image analysis of FUCCI time-lapse datasets, several semi-automated analysis tools exist in which the user is involved in a laborious procedure of either manually annotating, or aiding the machine by meticulously supervising a prediction for the cell location and cell cycle status [8–10]. Out of these, however, even the most recently developed CPU-based tool (FUCCItrack, [8]) is inadequate, as we show, to handle in an accurate and timely fashion the cell cycle analysis of thousands of extremely dynamic cells that collectively migrate in each field of view (FOV). For this case, a Graphics Processing Unit (GPU) acceleration is essential for performing efficient image analysis that combines biological cell segmentation followed by position tracking. The main benefit for this is that many image analysis tasks often scale linearly on a GPU, facilitating the processing of massive datasets in a timely manner. Moreover, nowadays, cloud servers provide accessible GPU hardware for leveraging modern deep learning-based image analysis techniques [11, 12].

**Fig 2:**
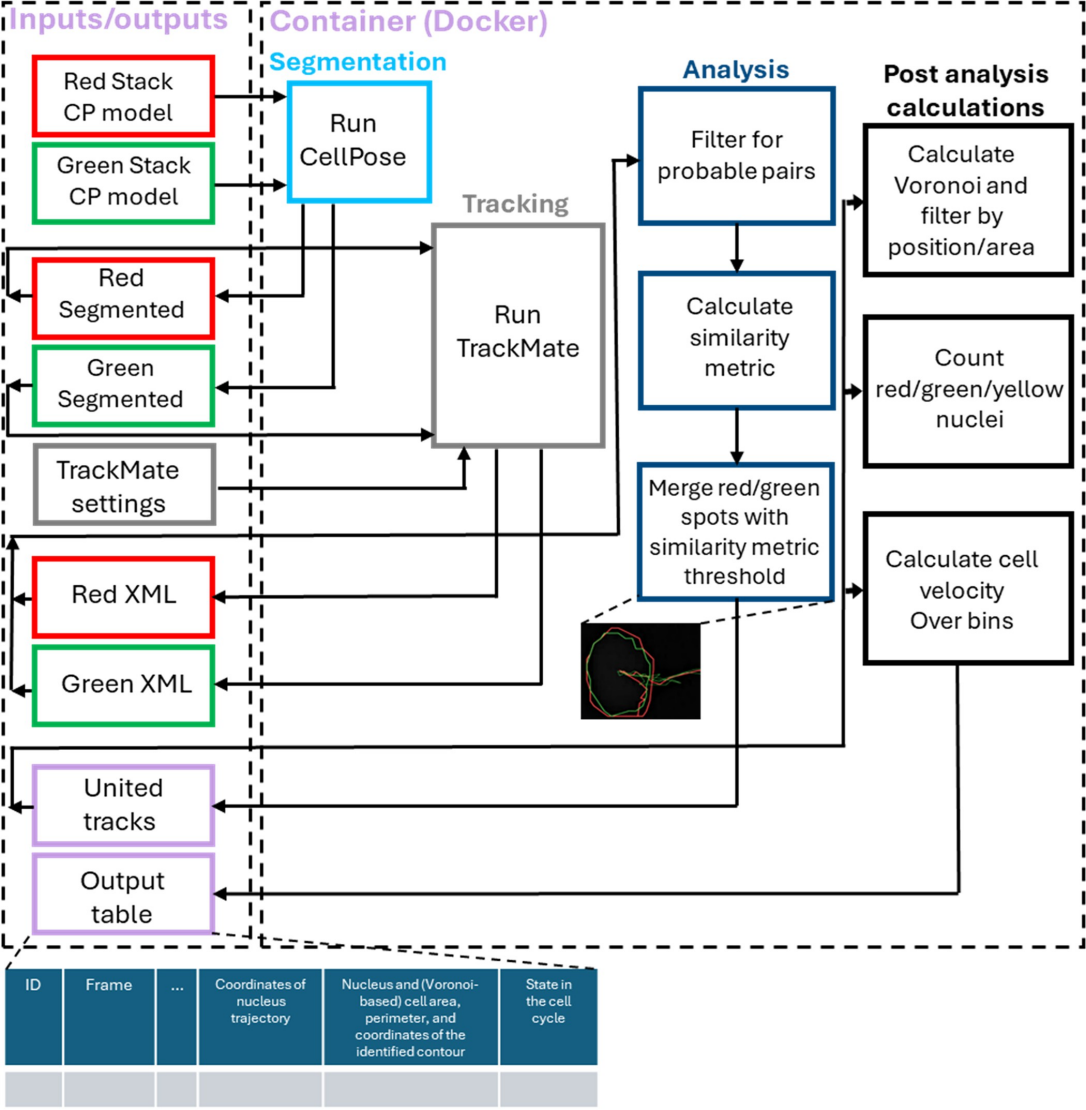
ConfluentFUCCI program flow chart. The flow chart is divided into two dashed rectangles. The left rectangle encircles the inputs and outputs of the program and each stage output becomes the input for the next stage. The right rectangle encircles all applications, custom codes, and processes, used by the program and are wrapped in a container using Docker [15].

Here we present ConfluentFUCCI, an open-source graphical user interface (GUI)-based framework. ConfluentFUCCI makes use of state-of-the-art Deep Learning algorithms to achieve accurate, fast and fully-automated analysis of cellular morphology, dynamics, and cell-cycle progression, for each FUCCI stained cell within thousands of collectively migrating cells. Our software uses an application programming interface (API) to match with Cellpose [13], an efficient Deep-Learning (DL) based segmentation tool with GPU acceleration, which facilitates accurate and flexible nuclear segmentation. Furthermore, embedded within ConfluentFUCCI is our new algorithm that: 1) interfaces with, and utilizes, a dynamic nuclei tracking module in TrackMate [14] that performs nuclei tracking in the two separate FUCCI fluorescent channels (Fig 1); 2) integrates tracking data from both channels, and assigns to each cell one continues track with compatible time dependent data that include cell and nucleus morphology, cell coordinates and their dynamics, and cell cycle state throughout the entire cell cycle. As such, ConfluentFUCCI leverages and integrates between two state-of-the-art tools that simplify the analysis of cell cycle data in dense migration assays, and defines a robust, and user-independent, criterion for G1 to S transition.

We will present a comparison with the most recently developed tool for such relevant analysis, and show that our computational scheme is more accurate than current solutions, applies to both sparse and dense cellular assays, and scales well to real-world conditions. We further show how we independently replicate the previously published data [4] of area-dependent cell-cycle transition in expanding tissue cultures of Madin-Darby canine kidney (MDCK) cells of epithelial origin.

## Materials and methods

### The presented approach for FUCCI signal analysis in dense and dynamic tissues

Until very recently the common approach to design a bioimage analysis pipeline was based on tools that are not well suited to the presented case here (yet, important for educational purposes–see supplementary paragraph 1 in S1 File) [8, 10]. In the case of multicellular migration, with thousands of cells per FOV, the FUCCI signal and its analysis raise multiple challenges. First, the nuclei are often clustered with potential overlapping in the image which frequently necessitates additional processing. Second, slightly out-of-focus regions in the image results in blurry nuclei boundary and difficulty in segmentation. Third, both MKO2 and mAG1 channels display fluctuating intensities in both space and time. Spatial fluctuations result from fluorophore inhomogeneity within a nucleus, cell to cell shape variability, and the extremely close proximity of the nuclei in collective migration experiments. All these features make the common threshold selection approach (supplementary paragraph 1 in S1 File) not suited for segmentation of all nuclei in the FOV and in each time point. Furthermore, temporal fluctuations in a dense tissue makes it difficult to simultaneously track nuclei that are in the G1 to S transition period, in both MKO2 and mAG1 channels using conventional approaches (supplementary paragraph 1 in S1 File). ConfluentFUCCI overcomes the above challenges and is now able to define the exact overlap window between the MKO2 and mAG1 illuminations, and thus gives the ability to declare the transition from G1 to S in a user independent manner. That is, for example, one can define the entire overlap window (yellow phase), or perhaps its mid time point, to be the G1 to S time of transition. That way, the conventional approach of declaring the G1 to S transition point based on specific fluorescent intensity value, and when the nuclei appears to the user as yellowish [4, 8], is avoided.

### Implementation and integration approach

A typical problem with an available code or algorithm for bioimage analysis in the literature are malfunctions that arise when the tool is used on different computational environments then it was originally designed in. These include different machines, operating systems, auxiliary folders and so on. For this purpose, we wrapped ConfluentFUCCI in what is known as container. Containers facilitate packaging of an entire runtime environment, including the specific versions of libraries, configuration files, and datasets [15]. Additionally, containers provide a stable platform protecting against potential numerical instability when running in vastly different computational environments. Finally, the adoption of containerization of scientific software provides a path towards scalability and compatibility with cloud computing platforms, thus allowing access to extensive computational resources, which are often required for analyzing contemporary bioimage datasets. Thus, ConfluentFUCCI can run on any operating system, with any type of available GPU or CPU. Yet, the biggest advantage we found in using a container was the ability to collect and integrate available state-of-the-art technologies such as deep learning, distributed computing, and modern webapps. Of specific interest to this work were NumPy (matrix operations) [16], Pandas (tabular data) [17], Dask (distributed computation) [18], Holoviews (graphs & figures) [19], Panel (WebApps) [20], Napari (multidimensional image viewer) [21], TrackMate (object tracking) [14], and CellPose (machine learning based segmentation) [13]. CellPose, TrackMate, and Napari, are core tools used within ConfluentFUCCI.

CellPose developers trained a neural network model (termed U-net) on a diverse set of cell/nuceli images to create a relatively generic approach applicable to a diverse set of imaging modalities and cell types. This is what commonly referred to as Deep Learning (DL), which requires huge amount of computation tasks that are best handled with a GPU. CellPose has a native support of executing on GPUs which can significantly speed up segmentation. In CellPose we used three FOVs of a dense confluent tissue for each red/green channel, and in 3 time points with increasing cellular density (and with a minimum of ~300 cells in each time point), to examine all available neural network trained models. We searched for the model that best segmented the FUCCI nuclei, prior to our further training, and found “cyto2” to be the most successful from all available models (including “nuclei”). We then set out to train new segmentation models based on “cyto2”, and used all 6 images to manually annotate all nuclei in each image. The training stage was performed using the default training parameters settings (“learning_rate”, “weight_decay” etc.) in CellPose. The results are the “red” and “green” models provided here. The models were able to detect FUCCI marked cells for different cell lines, and for different imaging systems (see *Time-lapse fluorescent and PC microscopy* paragraph).

TrackMate is a mature and performant cell tracking software, which is distributed as a FIJI plugin. While both tools are open-source, interfacing between Python and Java is neither straightforward nor error free. Furthermore, TrackMate is GUI driven and difficult to operate programmatically. We overcame this by packaging both Fiji and TrackMate, as an easy to use container. We designed and included in the container a custom-built middleware for loading an image stack, setting up tracking settings and saving the results. ConfluentFUCCI uses TrackMate to track all detected nuclei in both fluorescent channels simultaneously. It then calculates a similarity metric to identify which tracks (or part of tracks) overlap in space & time in both green and red channels. This lets us definitively merge individual red/green tracks into one continuous track throughout the cell cycle. For each such track all the available information is saved. This includes trajectory, area, shape, and cell cycle state.

Finally, to display FUCCI tracks and other relevant data we present the user with a GUI design adopted from Napari. Napari is a highly interactive and time efficient multidimensional image viewer, that is by itself based on prominent GUI-design and memory management environments. It is important to note that Napari enables some direct access to relevant image analysis tools, and although Napari is not ideally suited to work with Trackmate, it does offer alternatives such as Btrack [22]. We, however, found it ideal to integrate CellPose, TrackMate, and Napari, together with our core computations, in one fully packaged tool.

### Workflow description and overview of functionalities

Fig 2 describes the entire workflow of ConfluentFucci that is carried out in a fully automated manner from start to finish. The process begins with the two image stacks (red and green), the (neural network) trained model and its inputted settings for segmentation (phase contrast images are for presentation purposes only and are not necessary–see *Data preparation* paragraph in the supplementary chapter in S1 File). The segmented stacks are individually passed to TrackMate (plugin in FIJI), together with the specific required settings (under *basic_settings*.*xml* file in GitHub, see availability statement). Segmented nuclei are tracked to identify red tracks on the red channel and green tracks on the green channel and are outputted as two XML files (also available in the user library). Thus far, every stack (red\green) was analyzed by itself. The program now searches which tracks in the red channel and the green channel need to be paired and be stored as one continues track of one specific cell. This is made in the following two main programmatic steps. First, to alleviate calculation effort, the code filters the pairs that are more probable to eventually be identified as belonging to the same cell from all possible pairs of green and red tracks. To do so, the image is divided to a grid of n×n equal squares, and only a cell-R_i_ from the red tracks, and a cell-G_j_ from the green tracks, that passed through the same square in the grid, at any time throughout their trajectories, are considered a probable pair. Second, for each probable pair R_i_-G_j_ we calculate a similarity metric S_ij_, defined as the average Euclidean distance between the centers of R_i_ and G_j_ nuclei throughout their trajectories. A pair R_i_-G_j_ for which S_ij_ is smaller than $2\;\frac{pixel}{frame}$ (for our 10X imaging—typical for a collective migration experiment) is defined as belonging to the same cell. We find that the pre-filtering process in the two described steps works optimally for n = 10. The program now takes an overlay of the green and red channels, and based on all nuclei centers performs a Voronoi tessellation in order to approximate the area of each cell. The validity of this area approximation approach was previously demonstrated and shown to be accurate only in confluent and dense conditions [4, 23, 24], and can also be seen in Fig 6B. Furthermore, we used the tangential velocity vector available for each time points in each nuclei track and approximated the local multicellular velocity field. This is the average nuclei velocity, calculated within a square bin having a 1/10 of the FOV length, and assigned to a point in the center of the bin. The calculation is made for 181 points located on an equilateral triangular grid with horizontal and vertical spacing of a 1/20 of the FOV length. Such local velocity field is meant, among others, to aid the user in qualitative comparison to relevant velocity fields that are typically calculated using Particle Image Velocimetry (PIV) with phase contrast imaging [5].

### Data and parameters available in the GUI version vs underlying source code framework

At the end of the process in the GUI version the user gets a heat map of the local velocities and a table (Fig 2) with all identified cells, their united trajectories, nuclei areas and perimeters, Voronoi based areas and perimeters, pixel coordinates of the identified contour of each nuclei and (Voronoi based) cell, and the state in the cell cycle. Taken together, the described capabilities give ConfluentFUCCI users a unique approach to examine the relationship in space and time between cell cycle status, dynamic, and many morphological aspects of the cell, and its nucleus. We note that pre-filtering parameters are not adjustable in the GUI version of ConfluentFUCCI, but only in the source code.

### Installation and operation

**Fig 3:**
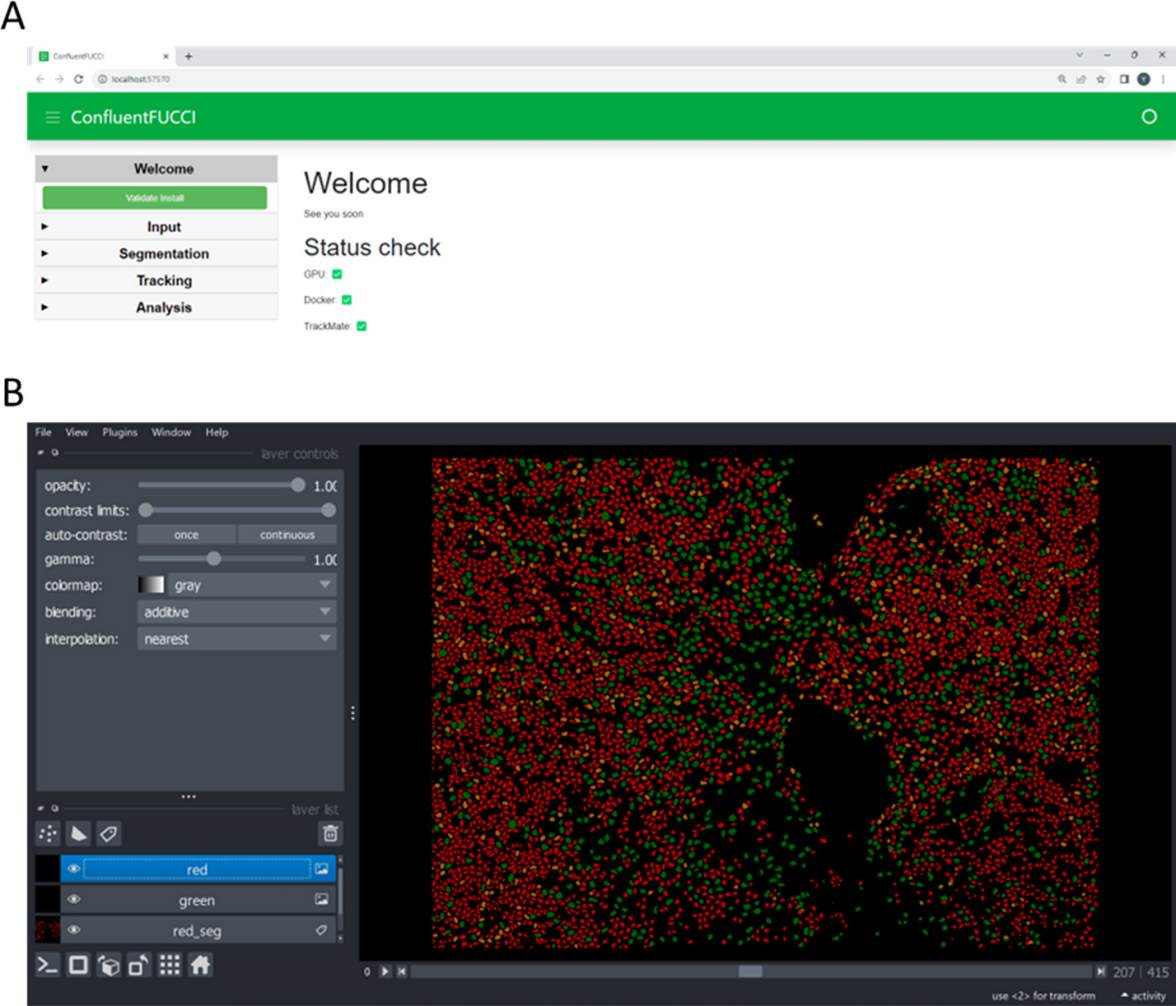
ConfluentFUCCI GUI. **a**, Initiation screen and, **b**, the viewer adopted from Napari (21). The user guide, with exemplary data files to work with, are available in the supplementary material. See also S2 Video.

ConfluentFucci is available to operate in open source code, but the quick and easier option is using the GUI version (Fig 3). The GUI is composed from the initiation screen, in which data is uploaded and initial settings are set, and the viewer screen which provides extremely easy and intuitive visualization of the segmented data. The installation and user guide are available in the supplementary material, along with S1 Video that follows the entire installation process.

### Cell culture

MDCK II cells, transfected to stably express the FUCCI transgene, were a kind gift from Dr. Lars Hufnagel [4]. cells were cultured in Modified Eagle’s Medium (MEM, MERK), supplemented with 10% fetal bovine serum (FBS, MERK), 1% penicillin/streptomycin (PEN-STREP) and 1% L-glutamine (Sartorius). Cells were incubated at 37C, 85% humidity and 5% CO_2_. A 500 μm inner wall insert (Ibidi) was placed in the middle of a 35mm diameter TC petri dish. 56,000 cells were seeded in each well of the insert (2 in total). Cells were incubated for 24h, after which the insert was removed from the dish, to create a wound like gap between 2 confluent layers.

### Time-lapse fluorescent and PC microscopy

**Fig 4:**
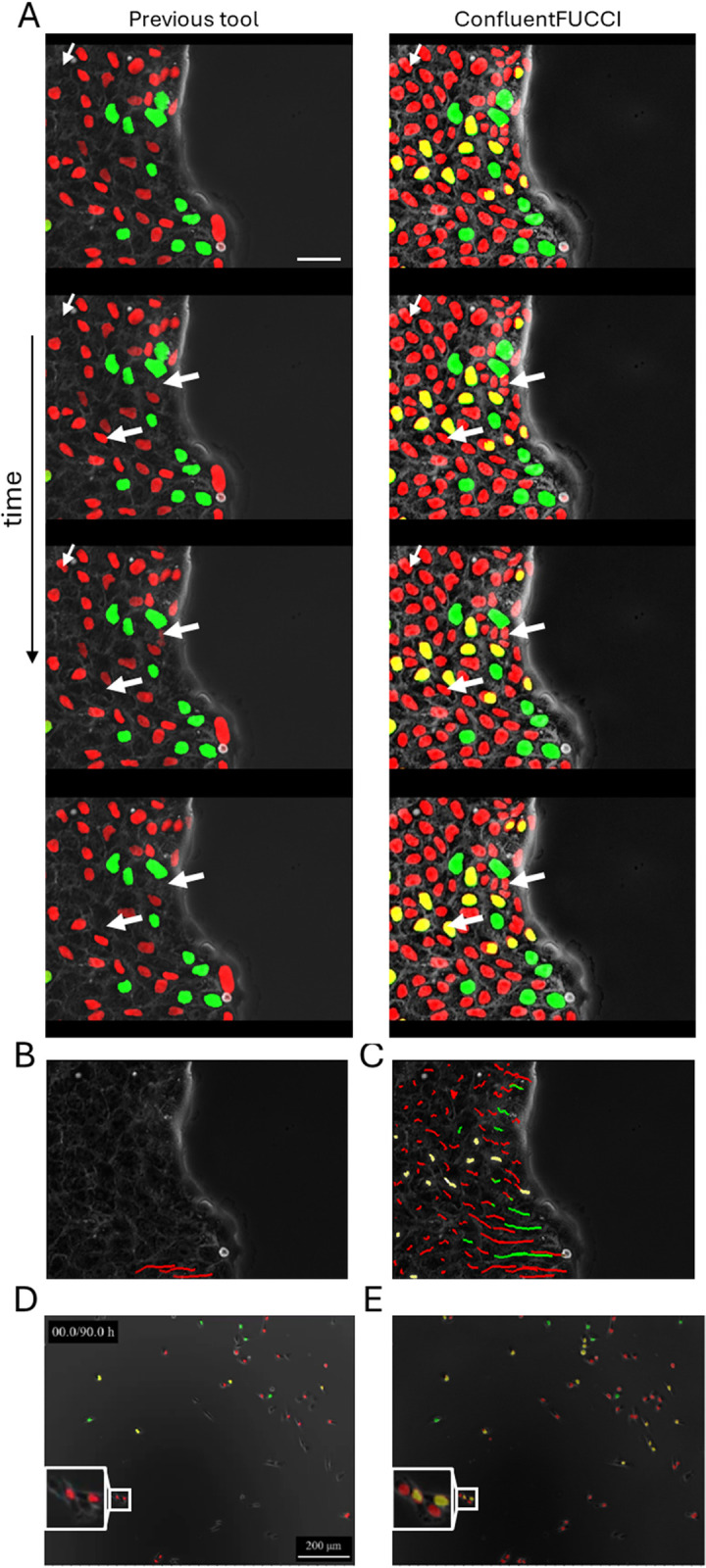
Robust segmentation and fully automatic time-tracking using ConfluentFUCCI. **a**, Confluent MDCK cells that collectively migrate in four sequential time points at 10 minutes intervals (see S2 Video). On the left, the segmentation done with the most recent published tool–FUCCItrack [8] which identifies only half of the population. On the right, the segmentation done with ConfluentFUCCI which identifies virtually all cells in the FOV. Thin white arrows indicate an instance in which a cell is identified at least 2 frames earlier in the right than in the left. Thick white arrows indicate a cell that is discontinuously identified on the left, while smoothly and continuously identified on the right. **b**, Three example of manually supervised tracking of cells (in **a)** using FUCCItrack, which took ~5 min of per track. **c**, Automatically generated tracks of all cells (in **a)** using ConfluentFUCCI, which took ~0.014 minutes of computation time per track. Scale bar (for a-c), 25 μm. **d,** identification and segmentation of FUCCI cells in sparse culture of MDA-MB-231 done using FUCCItrack [8] results in many missed cells, as opposed to the results obtained using ConfluentFUCCI. Blowup indicates misidentification of FUCCI markers in regions where cell density increases.

After insert removal, Petri dishes were secured under Zeiss Axio Observer 7 microscope equipped with an Axiocam506 MONO camera, and a stage top incubator maintained at 37C, 85% humidity and 5% CO_2_. On a 10X objective, 3 channel images were obtained (phase contrast, green and orange), every 10 minutes on a FOV with 2048X2048 pixels. Green channel was obtained with the 38 HE filter set (470/525, Zeiss). Orange channel was obtained with the 43 HE filter set (550/605, Zeiss). To comply with FUCCItrack accepted input [8], the data used in Fig 4A–4C was acquired with Leica DMI 8 microscope equipped with ORCA-Flash4.0 V2 camera, with the same imaging parameters (as the Zeiss system) and culture conditions, and a seeding density of 105,000 cells in each well.

## Results

### Robust and fully-automated segmentation and time-tracking during cellular collective migration

As mentioned above, several tools have been uniquely developed for image analysis of FUCCI time-lapse datasets, with the most recent and advanced one being FUCCItrack [8, 10]. Although this tool does make some progress in bridging the existent literature gap for FUCCI data analysis, it suffers from a few substantial drawbacks in the context of analyzing a dense tissue that collectively migrate. In FUCCItrack the segmentation process is based on the common approach of combining adaptive thresholding, area-based filtering, and the morphological closing operation (supplementary paragraph 1 in S1 File). We learned that this approach is indeed unsuitable for accurately segmenting and tracking FUCCI data in a dense tissue, after comparing the analysis performed by FUCCItrack vs. ConfluentFUCCI of our data of epithelial MDCK-FUCCI cells that collectively migrate. In a typical example shown in Fig 4 (raw data available, and shown in S2 Video), we observe three major differences between the two analyses. First, Fig 4A shows that within a single FOV the vast majority of cells are identified by ConfluentFUCCI as opposed to a substantial smaller fraction that is identified by FUCCItrack. A further quantified comparison is shown in S1 Fig in S1 File, where the parameters used in FUCCITrack are shown in S2 Fig in S1 File. Second, ConfluentFUCCI continuously detect all nuclei throughout the entire duration of the experiment (Fig 4A), whereas, FUCCItrack discontinuously detect many nuclei that flicker on and off (Fig 4A; white arrows). Third, all time tracks for nuclei positions are automatically produced by ConfluentFUCCI (Fig 4C). However, FUCCItrack requires the user to manually choose each single nucleus to be detected (i.e manual seeding), and furthermore to supervise the tracking process frame by frame (Fig 4B). We manually tracked multiple cells and measured ~5 minutes per track of labor time, which scale to a staggering time of ~8 hours for just this one position of highly sparse condition, in just one experiment. For comparison, we analyzed our data with ~11k cells, and the entire analysis took 2.5 hrs, which means ~0.014 minutes per track. We made further comparison for even sparse systems using available data previously analyzed with FUCCItrack [8]. Fig 4D shows a snapshot of a sparse culture of MDA-MB-231 cell line that is stably transfected for the FUCCI transgene. Interestingly, ConfluentFUCCI was able to detect all nuclei in the image, with FUCCItrack identifying less than half, and misidentification seemed to occur in high cell density locations (Fig 4D; blowup). We note that in FUCCItrack neither different manual seeding we made for tracking cells, nor any other different parameter settings, changed the comparison to ConfluentFUCCI results.

### Tissue-level monitoring for cell cycle status during collective migration

**Fig 5:**
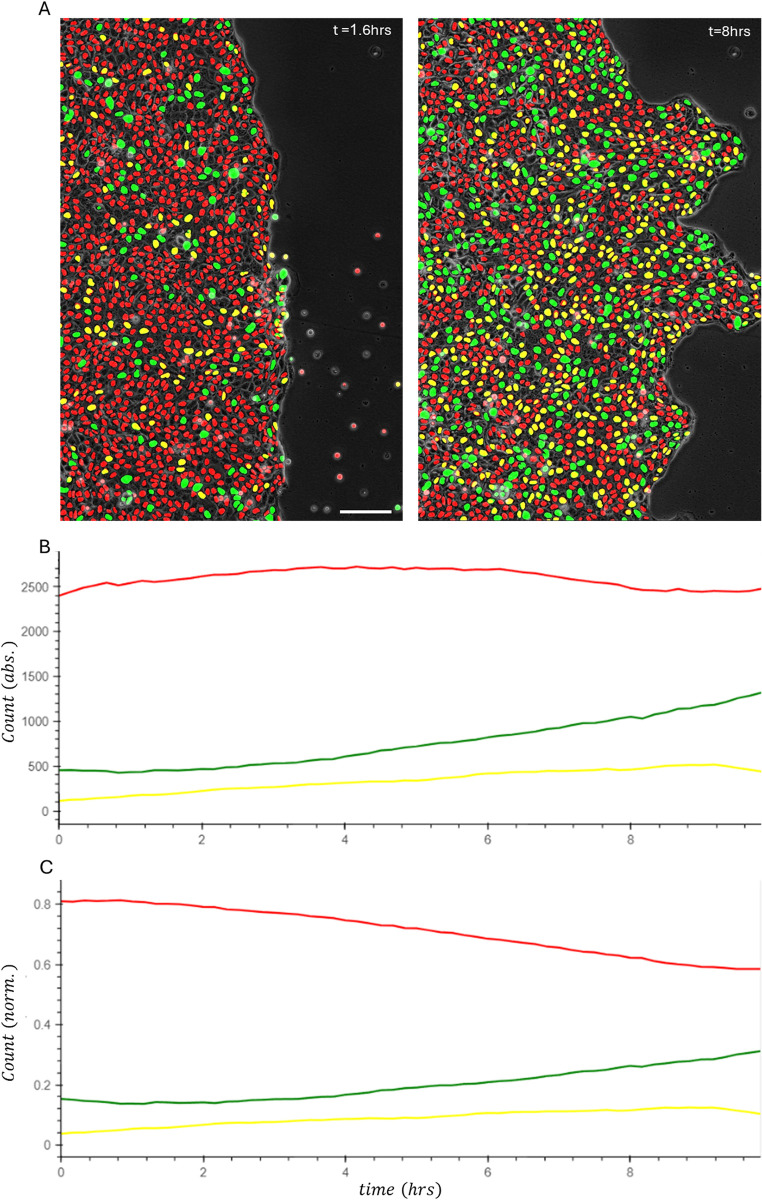
Tissue level monitoring of cell cycle progression during collective migration. **a**, Segmentation in two time points out of an analyzed stack of confluent MDCK cells that collectively migrate, post barrier lifting, for a 10 hours period. On the left t = 1.6hrs, and on the right, t = 10hrs. Raw fluercence data for the presented images is shown in S3 Fig in S1 File. Scale bar, 100 μm**. b**, absolute number, and **c**, normalized number (fraction), of red\green\yellow tagged cells at each time point.

The most straightforward use of the FUCCI system is to assess viability and global proliferation patterns at the tissue level [6, 25]. For example, monitoring the ratio of cells that transition to S state in one culture sample that was pharmacologically manipulated, or implanted and then extracted from in situ growth [26]. The FUCCI system can then provide a powerful method to evaluate cell cycle related mechanisms, with the most pronounced one being studies of cell cycle targeted drugs for cancer growth suppression and metastatic inhibition [27–29]. Famous metastatic hallmarks are the epithelial to mesenchymal transition (EMT), or partial EMT, and resultant cellular migration capacity [30–32]. Hence, even without considering single-cell level analysis, just a robust computerized tool that counts, per FOV, the number of cells in total, and number of cycling cells, may provide great clinical contribution. Fig 5A demonstrate the ability of ConfluentFUCCI to accurately segment nuclei, and identify cell cycle status, in virtually all cells in a collective migration experiment of MDCK-FUCCI cells throughout a 10hrs duration. Not like previous studies which intentionally design the culture for extremely low density and counts about a hundred cells per FOV [8, 10], in Fig 5B we show how the ConfluentFUCCI counts about three thousand cells per FOV. This gives a remarkable statistical power, and the ability to clearly define the global proliferation trend in the migrating tissue. Consistent with previous studies [4, 33], at early times the number of cells in total, and the number of cycling cells do not change (Fig 5B). This shows that the initial expansion of the tissue is dominated solely by collective cellular migration and not by proliferation. After about two hours of collective migration the fraction of non-cycling cells start decreasing, with a directly compatible increase of the fraction of cycling cells (Fig 5C). It is important to note that the described (Fig 5) cell cycle analysis at the tissue-level, is of course not limited to dynamic processes and can be used for fixed samples or histological sections with high cellular density.

### Single-cell-level monitoring for cell cycle status, dynamics, and morphology

**Fig 6:**
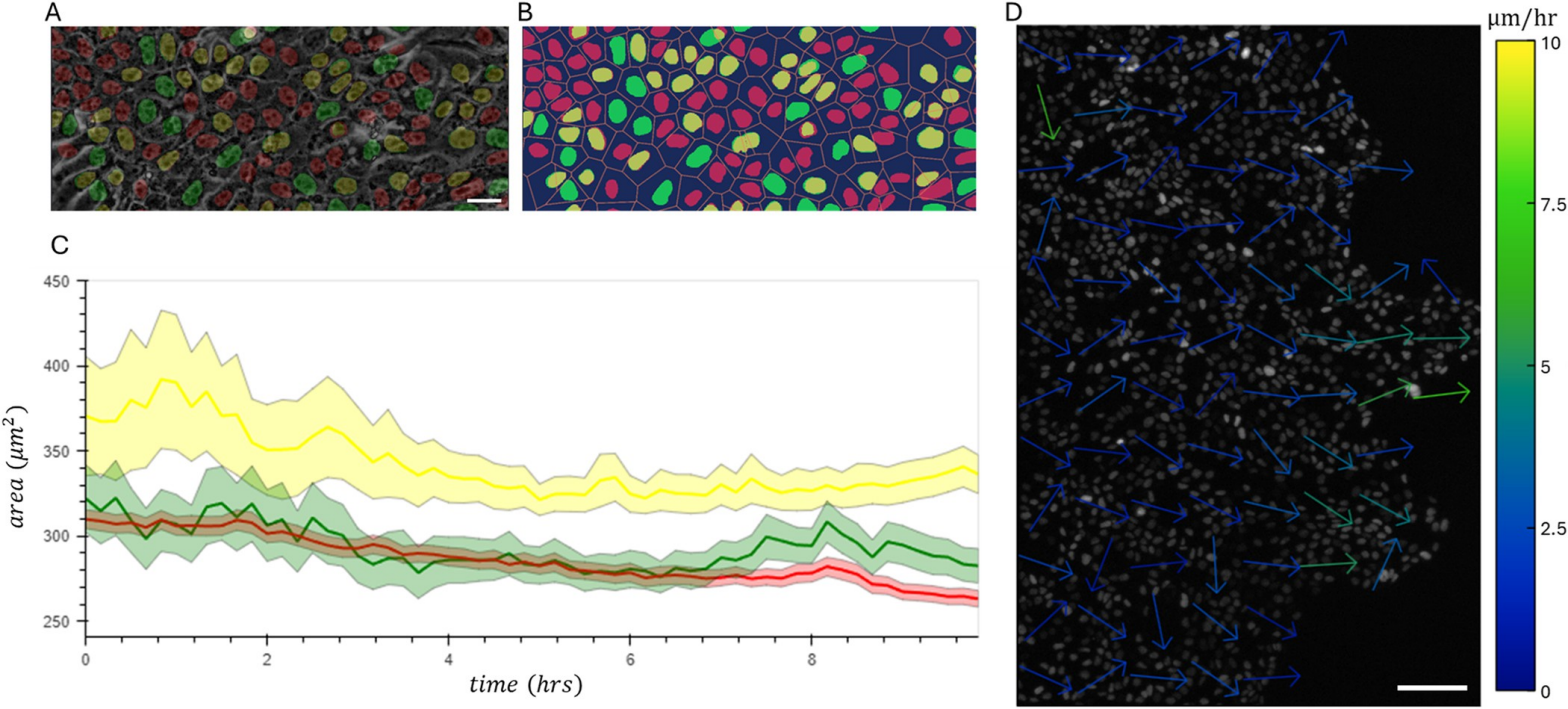
Single cell level tracking of all cells within an advancing tissue, that is confluent and dense, allows measuring cellular morphology and velocity field. **a**, Confluent MDCK cells that collectively migrate, post barrier lifting (as in Figs 4A and 5), with fluorescent FUCCI tags overlaid on the phase contrast (PC) image. Scale bar, 25 μm. **b**, The area of each cell is approximated using Voronoi tessellation that is based on nuclei centers. **c**, Solid lines, colored according to cell cycle state, show the average area of cells in each state. Colored shaded regions indicate 95% confidence intervals. **d**, Velocity vector field based on nuclear velocity. Scale bar, 100 μm.

The regulation of cell cycle progression by either intracellular biochemical cues, or intercellular mechanical cues, has been extensively studied for many years now [4, 5, 34, 35]. Nevertheless, when it comes to cell cycle regulation during tissue expansion and cellular collective migration, it is still unclear what are the main contributing molecular or mechanical factors. Many key mechanical factors, such as intercellular stress, cell morphology, or growth rate, are inherently related to sterical constraints imposed on each cell by its multicellular neighborhood. Unlike current tools [8, 10], ConfluentFUCCI allows for a robust and fully automated monitoring of all nuclei during collective migration and enables investigating the spatio-temporal connection between cell cycle progression and a variety of participating mechanical factors. To demonstrate such investigation, we decided to explore the relationship between cell area and the transition from G1 to S state in collectively migrating epithelial cells. Fig 6A and 6B shows how the condition of a condensed cellular environment allows for a good size estimation of each cell using Voronoi tessellation that is based on the nuclei positions. Fig 6C shows that the area of cells that are transitioning from G1 to S are systematically bigger and thus replicates previously published data [4]. ConfluentFUCCI also allows to go further and explore the relation between proliferative patterns and multicellular motions. In this context, Fig 6D shows the velocity field, which is possible to calculate only because ConfluentFUCCI tracks all cells in this high cellular density conditions, and in essence transform Lagrangian trajectories to an Eulerian velocity field.

## Discussion

This work presents a new fully-automated image analysis tool for analyzing live fluorescent markers for cell cycle monitoring (FUCCI), in time-lapse microscopy data of cells that collectively migrate on a flat surface. The tool is capable of accurately segmenting and tracking nuclei in dense tissues and identifying the cell cycle stage of individual cells. In comparing ConfluentFUCCI with the most recent and relevant existing tool, we found that ConfluentFUCCI outperforms it in terms of nuclei segmentation ability, accuracy, and time tracking, both in sparse and dense environments. Our work also demonstrates the ability of ConfluentFUCCI to monitor cell cycle status and dynamics at both the tissue and single-cell levels, as well as replicate the main features of the previously published relationship between cell cycle progression and cell size in the first ten hours of collective migration [4]. That study implied that a mechanosensitive checkpoint ensures that only cells big enough, and that can physically facilitate all mitosis related events, will progress in the cell cycle. To establish the existence of that area related checkpoint in greater depth one needs to further examine the correlation between the transition event to other, molecular, or physical, temporal features of the transitioning cells. Although such investigation is beyond the scope of this work, many of the needed to be explored physical features, during G1 to S transition, can be investigated by ConfluentFUCCI and include, for example, area (and contour coordinates) of each cell and its nucleus, trajectories, and the Eulerian-like velocity field in the layer. But, of course, not only physical factors can affect features of cell cycle progression, but also genetic predisposition. Such a study on heritable cell-cycle-influencing factors was previously done with a robust computation tool that used a Histone marker to analyze nuclear structural changes and identify different stages in the M (mitosis) state [22]. Although not designed to identify the G1 to S transition, one can certainly use this tool in combination with ConfluentFUCCI to study heritable influence on G1 duration time. All in all, we suggest that ConfluentFUCCI can provide valuable, reproducible and user independent insights into cell cycle regulation in dense cellular condition in the context of various biological disciplines including cancer growth and metastasis. We would like to note that ConfluentFUCCI was developed as part of a current effort we take to integrate, on a large scale of data, measurements of cell cycle dynamics, ECM-cell and cell-cell mechanical stresses, in different crowded cancerous environments.

## Supporting information

S1 File (DOCX)

S1 Video (MP4)

S2 Video (AVI)
