# Supplementary material for: ConfluentFUCCI for fully-automated analysis of cell-cycle progression in a highly dense collective of migrating cells
Source: PLoS One. 2024 Jun 26;19(6):e0305491. doi: 10.1371/journal.pone.0305491 (PMC11207131; doi:10.1371/journal.pone.0305491)

**Supplementary Material**

**Figure S1** – **Quantified comparison between FUCCITrack and Confluent FUCCI**. Since in the context of this work no relevant FUCCI-benchmark data is available in the literature, and since ConfluentFUCCI identifies virtually all nuclei in every given image (Fig. 3-6 in the main text), we defined ConfluentFUCCI data as the ground truth to be compared to. The comparison was made on a 60 frames FOV of ~2000 collectively migrating MDCK cells. **a**, For proper visualization a sub-window of the FOV shows the FUCCI nuclei in two time points as identified by ConfluentFUCCI (top) and FUCCITrack (bottom). Scale bar, 50 μm. **b**, The top panel shows the proportion of identified FUCCI nuclei in FUCCITrack compared to ConfluentFUCCI in both red and green channels as a function of time. The bottom panel shows the average proportion with a 95% confidence interval (based on a 60 frames population).


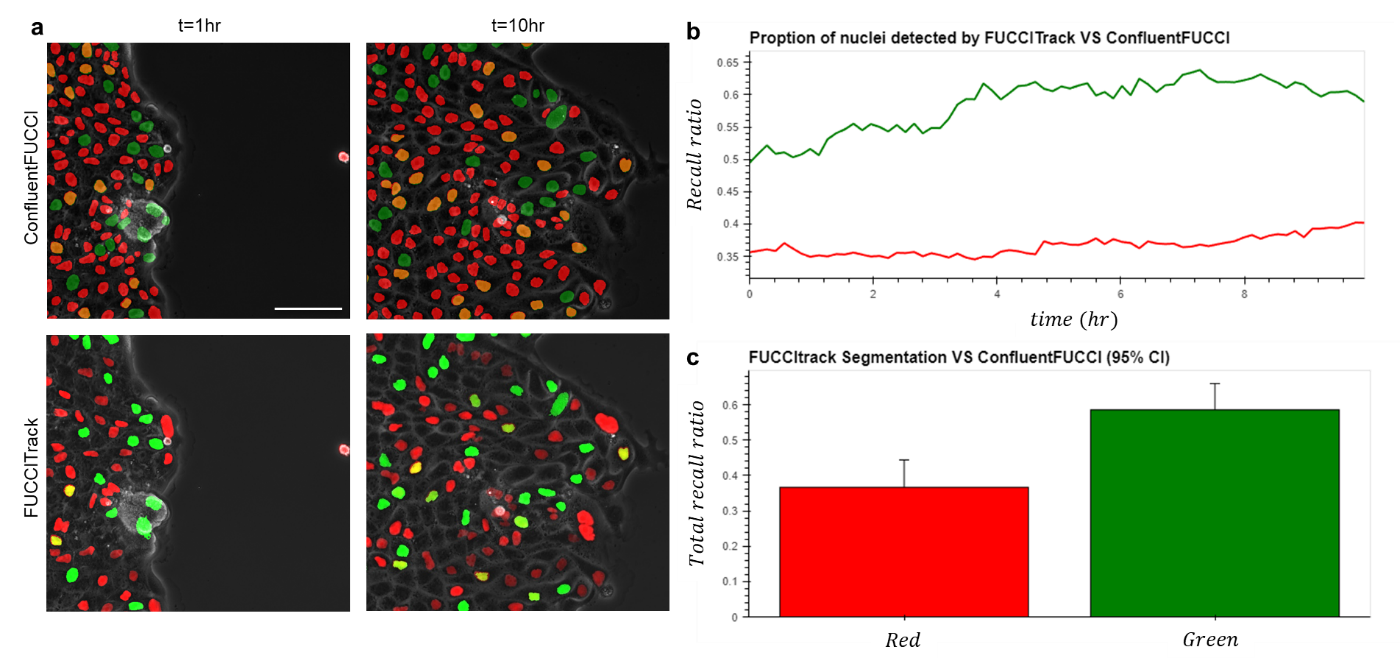


**Figure S2** – **Parameters used in FUCCITrack for comparison with Confluent FUCCI**. There were only two tabs, “data loading” (**a**) and “collective” (**b**), used to run the analysis in FUCCITrack. We used the default parameters selection in FUCCITrack (a&b) in all comparisons shown throughout the entire text.


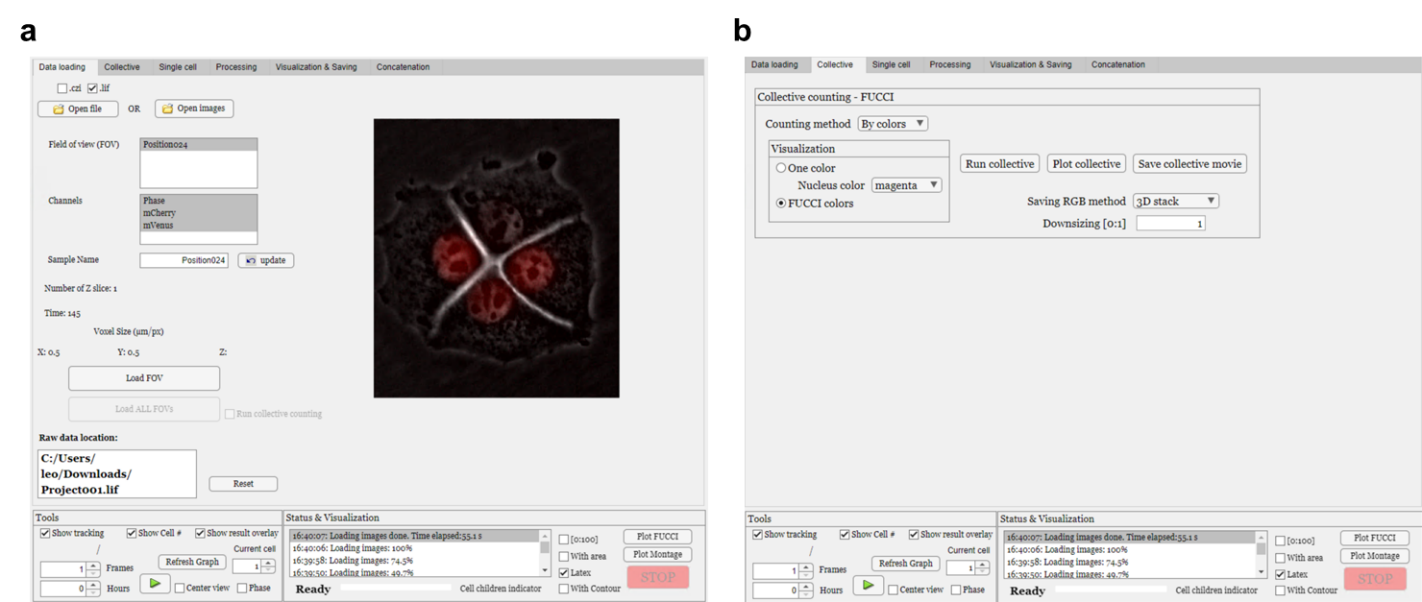


**
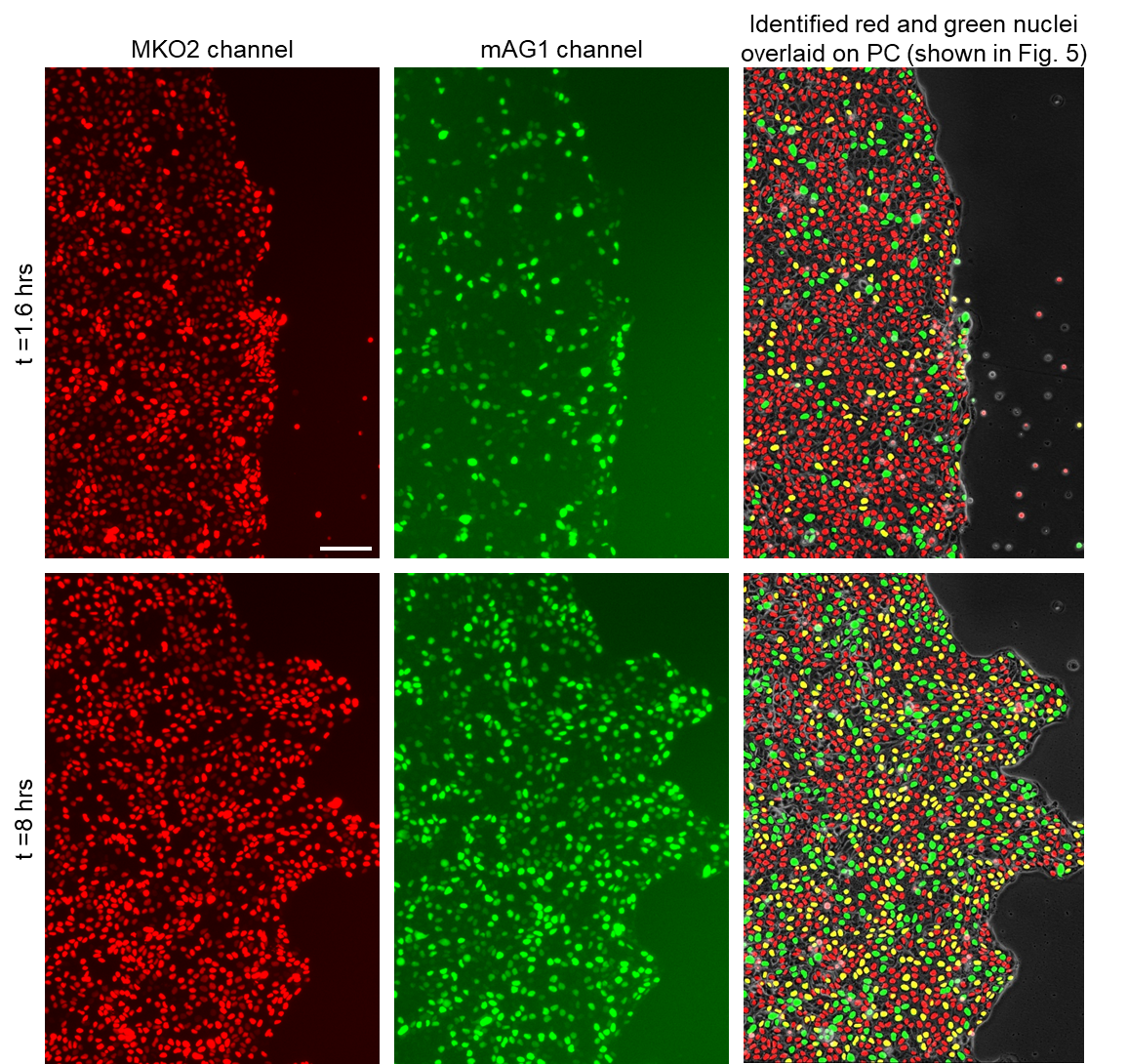
**

**Figure S3** – Raw FUCCI fluorescence data shown in Fig. 5. Scale bar, 100 μm.

**Supplementary paragraph 1: The common image analysis approach we avoid**

Until recently, the image analysis pipeline for biological cell shape and dynamic analysis typically involved a series of manual and semi-automated steps. The process began with acquiring images of cells using a microscope, followed by image preprocessing to remove noise and enhance contrast. Techniques such as Gaussian blurring and median filtering were commonly used to reduce noise and improve the quality of the images. Then, to separate individual cells from the background the three main classes of techniques that were employed included thresholding, edge detection, and region growing. Thresholding involves setting a threshold value to separate the foreground from the background in an image. Edge detection involves identifying the boundaries of cells by detecting changes in intensity between adjacent pixels. Region growing involves selecting a seed pixel and iteratively adding adjacent pixels that meet certain criteria to form a segmented region. Combined with these techniques, known morphological operations, such as erosion, dilation, and opening, were commonly employed. These were used to remove small objects or fill gaps in segmented regions, and to produce a finalized segmented image. To track a specific cell, or a segmented region, from one time point to another, a variety of tracking algorithms were used. These include algorithms such as nearest-neighbor, and Kalman filter, that relied on feature matching between frames to establish cell identity and its dynamics.

It is important to emphasizes that most mentioned techniques were often time-consuming, labor-intensive, prone to human errors, and did not scale well with large datasets. Hence, it was in the hands of the experienced bioimage analysts to creatively tailor from all mentioned techniques an image analysis pipeline that in most cases was suitable for a specific set of images, taken in a specific experiment, by a specific lab. Since we aspire to provide a robust tool to analyze the FUCCI data in a variety of flat culture conditions, the above approach that was commonly taken before [8, 10], does not suffice.

**ConfluentFUCCI – a user guide**

With ConfluentFUCCI we aim to give the end user a comprehensive, self-contained, easy to use tool for time-lapse analysis of cells transfected with the FUCCI system.

We provide two ways of installing the tool:

- Manually via the Python packaging tool with `pip install confluentfucci` (available on github repository)
- Container – the recommended option: See supplementary video 1.

**System requirements:**

- Python 3.10 (only for manual installation)
- Docker
- Optional: GPU + CUDA drivers (for speeding up segmentation) – highly recommended for non-trivial datasets. Examples of typical run times in different hardware for segmenting and analyzing data sets with varying cellular densities are given in supplementary table 1.

| FOV dimensions (X·Y·#Frames) | # Cells | Hardware | Segmentation (minutes) | Analysis  (minutes) |
| --- | --- | --- | --- | --- |
| 2048x2048x145 | ~2k | Setup 1 | ~30 | ~180 |
| 2048x2048x145 | ~2k | Setup 2 | ~240 | ~180 |
| 1212x342x60 | ~70 | Setup 1 | ~1.5 | ~0.25 |
| 1212x342x60 | ~70 | Setup 2 | ~12 | ~0.25 |
| 1212x342x60 | ~70 | Setup 3 | ~22 | ~0.5 |
| Hardware:  Setup 1 - Ryzen 5950X 16 Core; Memory 131 GiB; GPU RTX 3090 24 GiB VMem  Setup 2 - Ryzen 5950X 16 Core; Memory 131 GiB  Setup 3 - Lenovo IdeaPad 3 15ALC6; AMD Ryzen 3 5300U 4 Core; Memory 5.63 GiB | | | | |

**Supplementary Table 1 –** Typical run times in different hardware.

Notice that some users have experienced issues when installing [Docker Desktop](https://docs.docker.com/desktop/install/windows-install/) or the underlaying [Windows Subsystem for Linux](https://learn.microsoft.com/en-us/windows/wsl/install) (WSL). Depending on your environment, you might encounter errors when installing or starting Docker Desktop. A working Docker install is required for using ConfluentFUCCI. Both the containerized and manual installs make use of docker to run some/all of the capabilities.

To simplify first time installation, we recommend you first install/update your WSL installation as detailed in multiple online guides (for example [this simplified guide](https://ubuntu.com/tutorials/install-ubuntu-on-wsl2-on-windows-11-with-gui-support#2-install-wsl)) . After making sure WSL works for you (for example, by [starting a Linux Distro](https://ubuntu.com/tutorials/install-ubuntu-on-wsl2-on-windows-11-with-gui-support#3-download-ubuntu)), install Docker Desktop and restart your machine. After restart and starting Docker Desktop, you should be able to execute `docker run hello-world` without an error via a CMD screen as seen in Figure S4:

**Figure S4 –** Running the command ‘docker run hello world on a CMD screen.


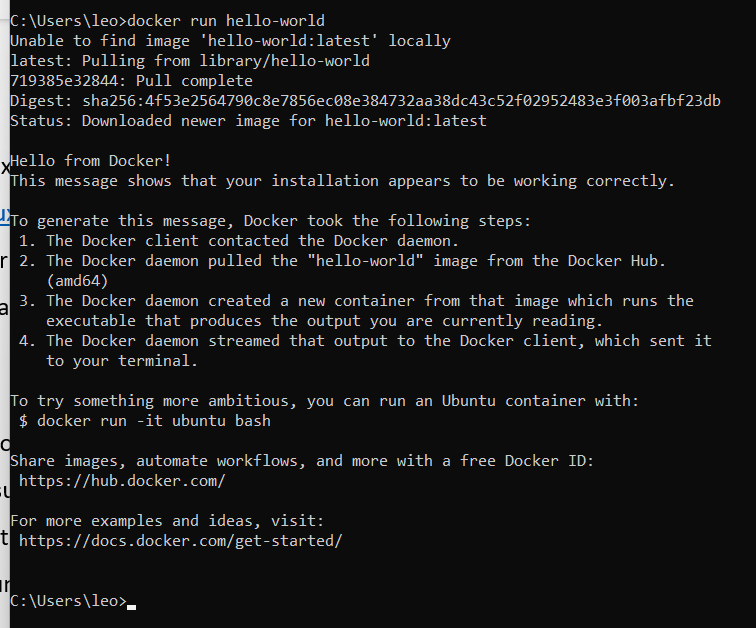


**Installation instructions for the recommended option:**

We provide a [docker-compose.yaml](https://github.com/leogolds/ConfluentFUCCI/blob/main/containers/confluentfucci/docker-compose.yaml) to simplify installation (reproduced below):

services:

confluentfucci:

image: leogold/confluentfucci:latest

# env_file:

# - .env

ports:

- "8080:8080"

- "9876:9876"

volumes:

- /var/run/docker.sock:/var/run/docker.sock

# Uncomment below to mount user data

# - "${DATA_PATH}:/data/user_data"

# - "${MODEL_PATH}:/data/models/user_models"

# Uncomment below to enable GPU (CUDA) acceleration on compatible devices

# deploy:

# resources:

# reservations:

# devices:

# - driver: nvidia

# count: 1

# capabilities: [ gpu ]

Open a text file, copy the above code lines, and save this file locally on your computer with this precise name '[docker-compose.yaml](https://github.com/leogolds/ConfluentFUCCI/blob/main/containers/confluentfucci/docker-compose.yaml)’. Alternatively, download the file from Github repository. Open a CMD screen, and change directory to where you saved the above file. In the same CMD screen, run `docker-compose up` (or `docker compose up`). This will bring up the ConfluentFUCCI application.

We expose two ports to serve the app:

- 8080 – the main GUI for the application
- 9876 – secondary GUI for displaying segmentation, Voronoi & tracking results

**Launching ConfluentFUCCI:**


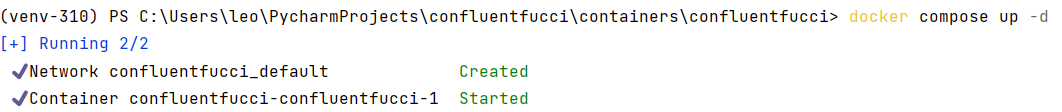
Open <https://localhost:8080> a few minutes after `docker-compose` reported the application is started, as you see below:

You should get to a page shown in Fig. S5. The menu on the left allows navigation in ConfluentFUCCI . Follow the buttons at the left of the screen, for a complete analysis.


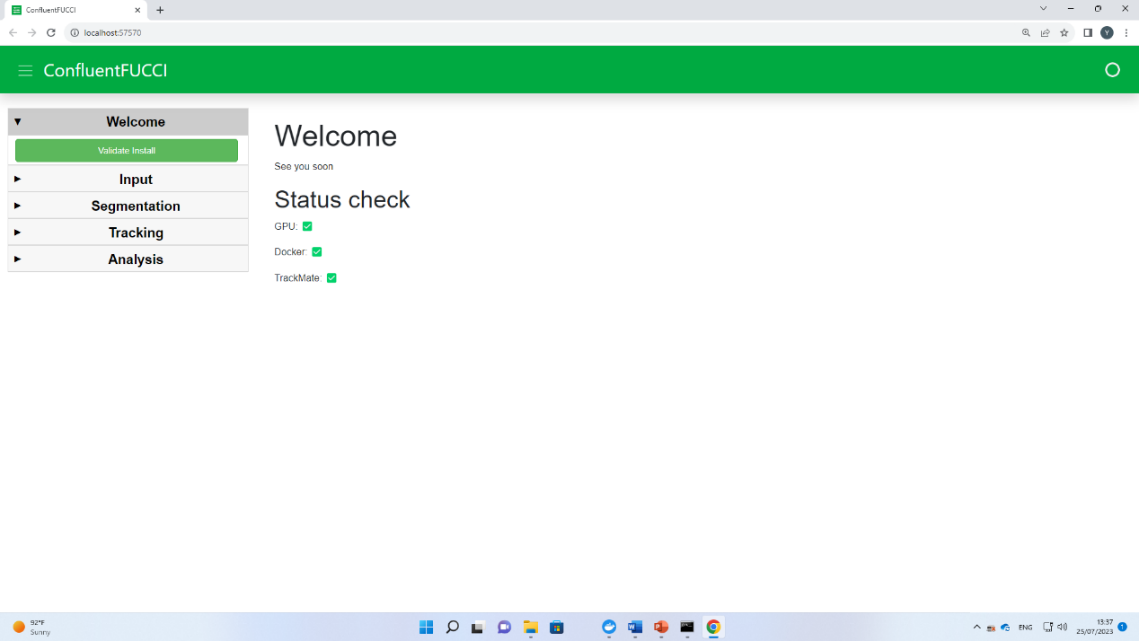


**Figure S5 – ConfluentFUCCI start screen.**

**Welcome screen:**

Upon launching, ConfluentFUCCI validates its own installation.

In the case that the GPU status check failed – we rely on CellPose and in turn on PyTorch, a machine learning library, for segmentation. We note that PyTorch supports running on a CPU but this mode is significantly slower. If you are running on a machine with a [CUDA capable GPU](https://developer.nvidia.com/cuda-gpus), and have the relevant drivers installed, you can leverage it to speed up segmentation. To do so, uncomment the last section in the above `docker-compose.yaml` file.

**Input screen:**In this section, please select your data and CellPose models. You can use the models we provide, or models you trained. For information on how to train your own models, please refer to the [CellPose documentation](https://cellpose.readthedocs.io/en/latest/gui.html#training-your-own-cellpose-model)**.**

**Data preparation:**

Your data should include three channels: two fluorescent FUCCI channels and one phase contrast channel. We emphasize that the system searches for 3 files, yet the phase contrast micrographs are for presentation purposes only. If you do not have a phase contrast, but instead any other micrograph you wish to overlay the identified FUCCI signal on, just name it “phase” and use it. If you wish to display only the fluorescent FUCCI channels, prepare a corresponding blank series of images, name it “phase” and use it.

Note that although more advanced FUCCI systems with more than two fluorophores are available, ConfluentFUCCI is constructed only for a 2-color system. Red is the general name for the G0/G1 label, while green is the general name for the S/G2/M label.


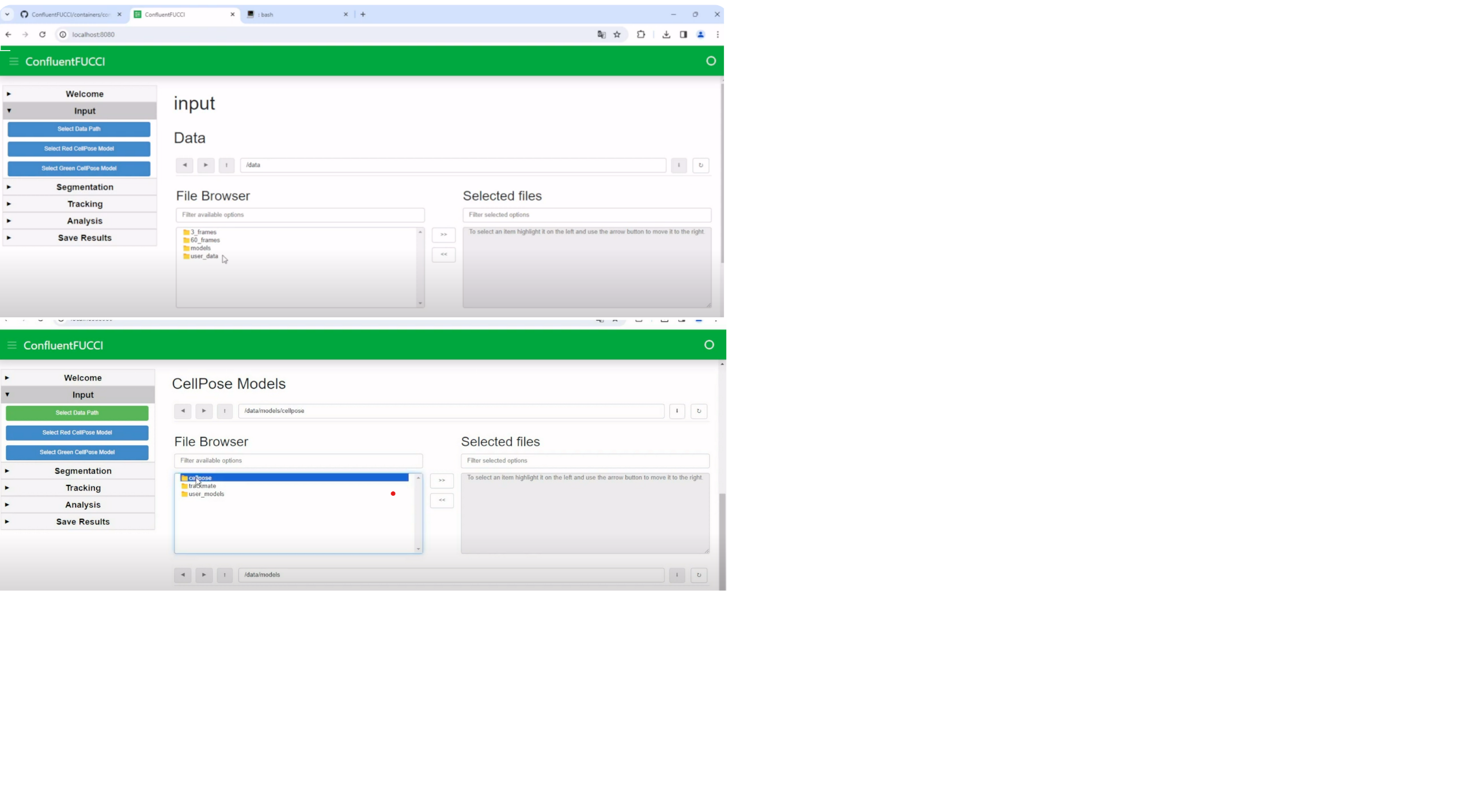


**Figure S6 – ConfluentFUCCI start screen.**

Please name the stack from the different channels as ‘red.tif’, ‘green.tif’ and ‘phase.tif’, respectively, with all 3 stacks saved at the same folder.

Press the ‘Select Data Path’. Browse the file explorer to enter the path to your data (Fig. S6).

Press the ‘Select Red CellPose Model’. Browse the file explorer to enter the path to your red CellPose model (Fig. S6).

Press the ‘Select Green CellPose Model’. Browse the file explorer to enter the path to your green CellPose model (Fig. S6).

The pretrained models are included in the installation.


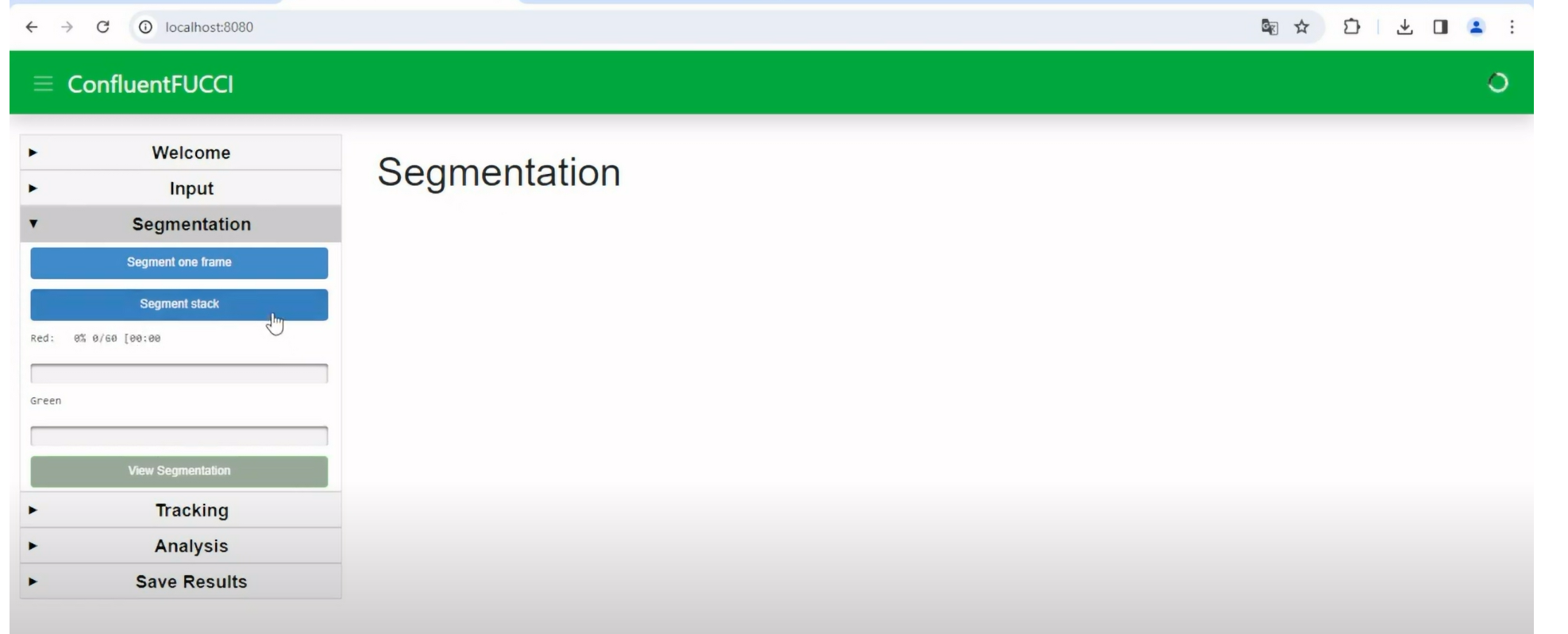


**Figure S7 – Segmentation screen. Progression bars: % of stack that was analyzed, no. of current frame/total number of frames, [time passed<remaining time, speed]**


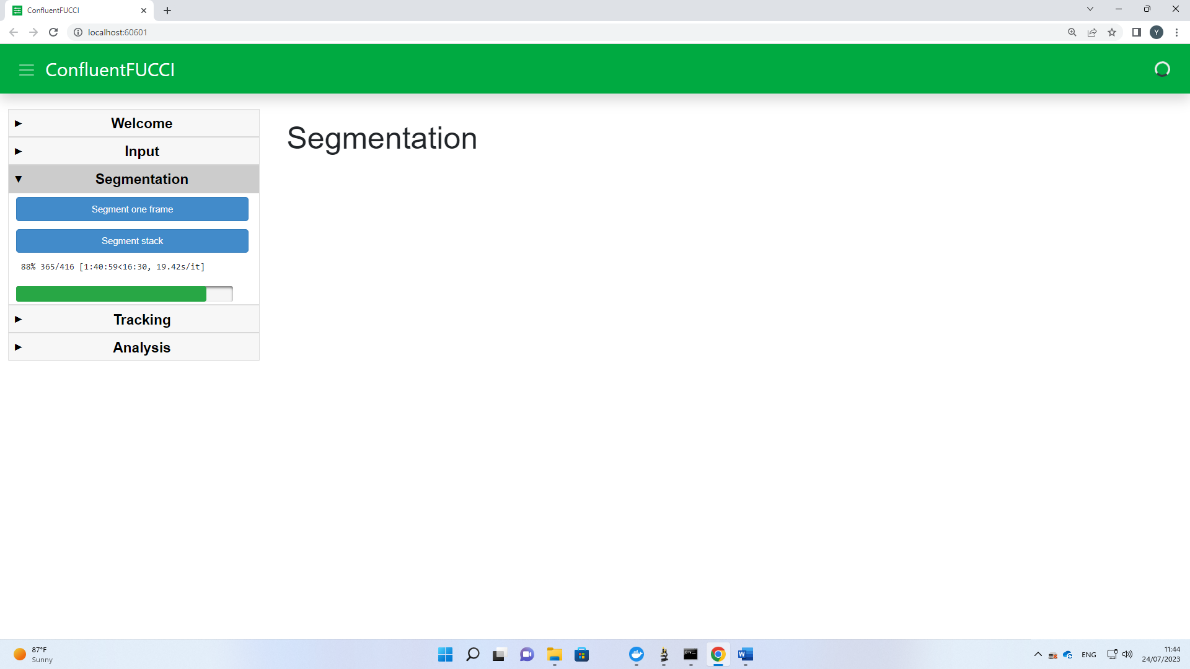


**Segmentation screen:**

Before running segmentation on the entire stacks, please run the segmentation on one exemplary frame only ('Segment one frame'), in order to check the validity of the segmentation. You can follow the progression of the segmentation with the progression bars (one for each fluorescent stack) that will appear under the 'Segment stack' button when you press it (Fig. S7). Once the segmentation is done, a viewer will open (see viewer section) on <http://localhost:9876> that will allow you to observe the segmentation results. It is recommended to repeat this with several frames in different times point.

You can now run a segmentation of the entire stack ('Segment stack'). This will segment both your fluorescent stacks (but not the phase). This may take a while, depending on the size of your data. For example, a FOV of 4096x3008 pixels in size, that covers a confluent layer with our chosen initial seeding density and image settings (as described in the methods section) will take about 13 to 20 seconds per frame on a RTX 3090 GPU.

**Tracking screen:**

Note that the viewer window must be closed before tracking is initiated. Press the 'Track' button. The tracking progression will be displayed in the black screen (Fig. S8). When tracking is done, you can open the viewer window at <http://localhost:9876>.

**Figure S8 – The tracking screen. Progression is reported by TrackMate in the black window.**


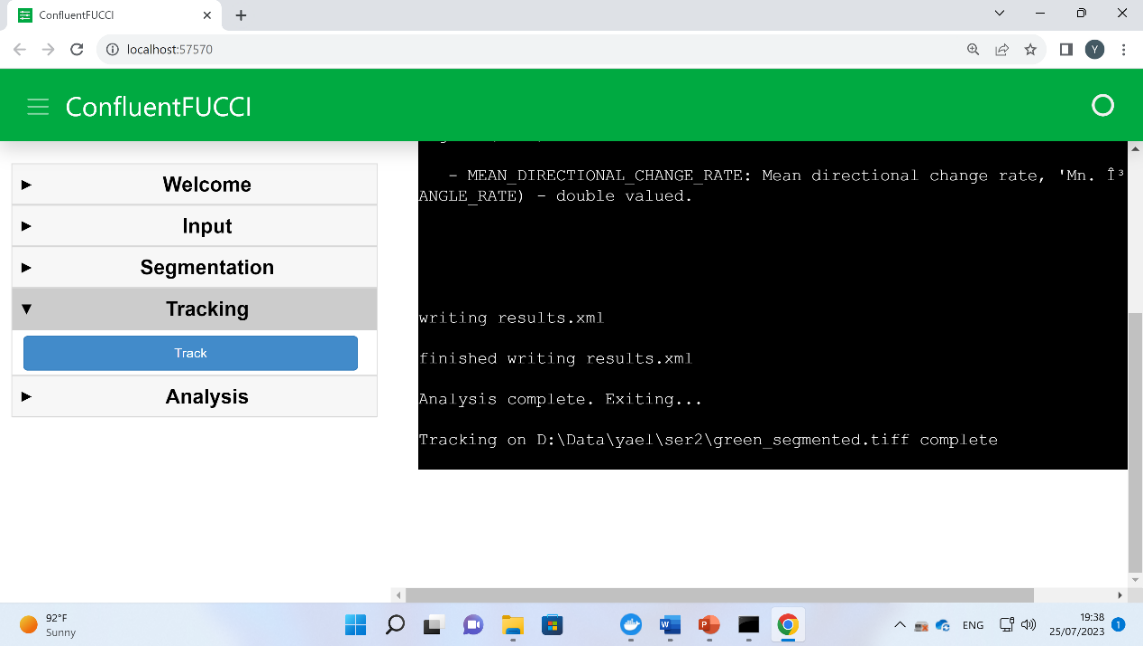


**Analysis**:

Before starting the analysis, you will be asked to close the viewer window again. After analysis is over, you can once again open the viewer window at <http://localhost:9876>.

During analysis, ConfluentFUCCI calculates a similarity metric (see detailed description in the method section). You can follow the progression of the metric calculations on the CMD screen (Fig. S9). This is a time-consuming process, and thus analysis may take a while (several hours), depending on the size of data. For example, a FOV of 4096x3008 pixels in size, that covers a confluent layer with our chosen initial seeding density and image settings (as described in the methods section) will take about 90 seconds per frame.

**Figure S9 – the CMD appearance during the calculation of the metric. The last line reads: %of completion |progression bar| number of tracks couples analyzed/total number of couples [time elapsed<time remaining, speed].**


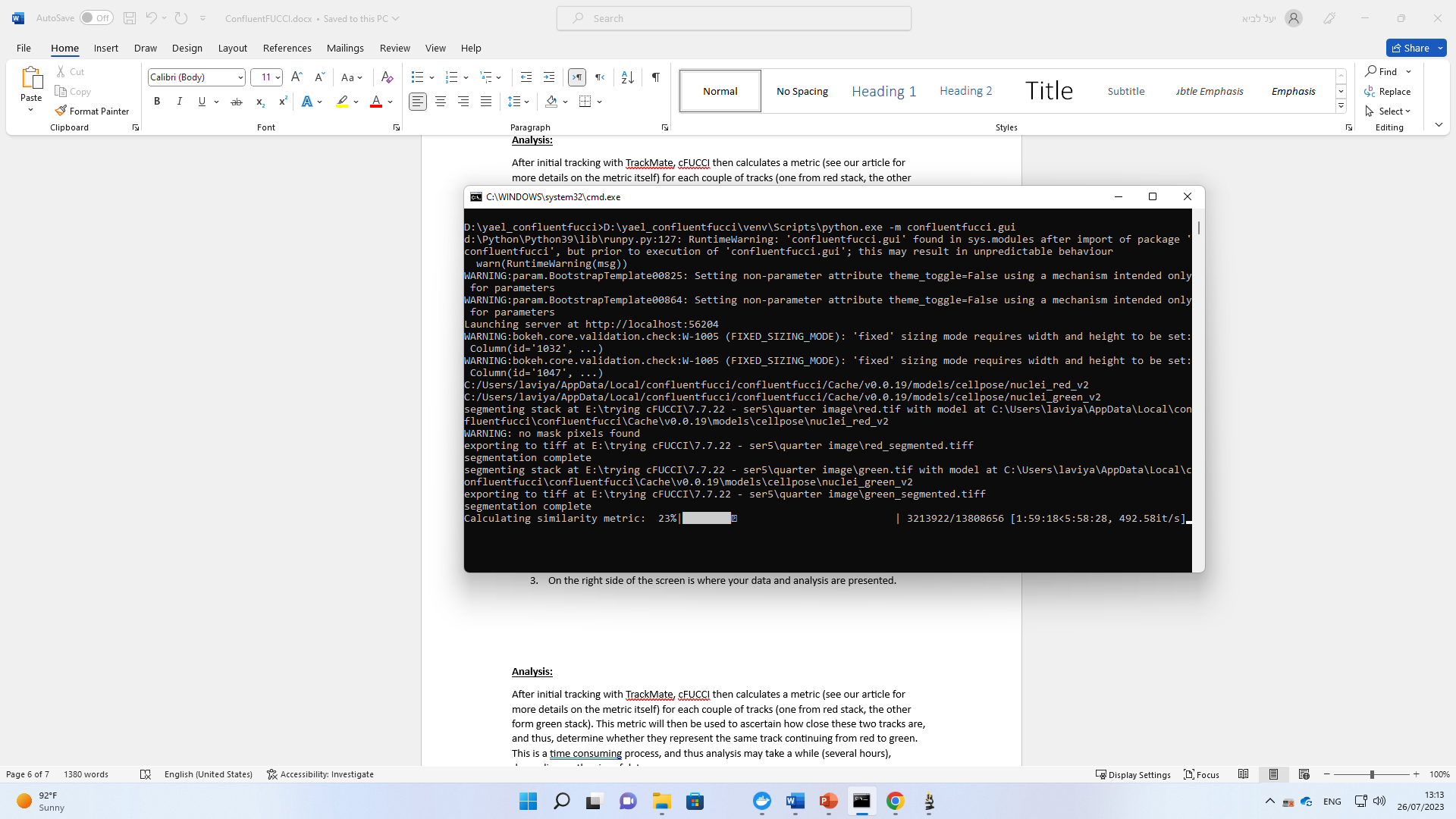


**The viewer**:

After finishing either tracking, segmentation, or analysis, open the ConfluentFUCCI viewer window at <http://localhost:9876> (Fig. S10).

In this viewer you can see the analysis results (segmentation, tracking, Voronoi tessellation) displayed upon your raw data. Here you can test the validity of the segmentation, see tracks from both fluorescent channels and other results.

The viewer is divided into 3 sections:

1. On the top left side of the window you can control the appearance of your raw data.
2. On the lower left side of the window you can choose what raw data (red stack, green stack, both) will be displayed, and what results will be displayed on your raw data (segmentation, tracking, analysis).
3. On the right side of the screen is where your data and analysis are displayed.

**Figure S10 – ConfluentFUCCI viewer,** adopted from Napari (21).


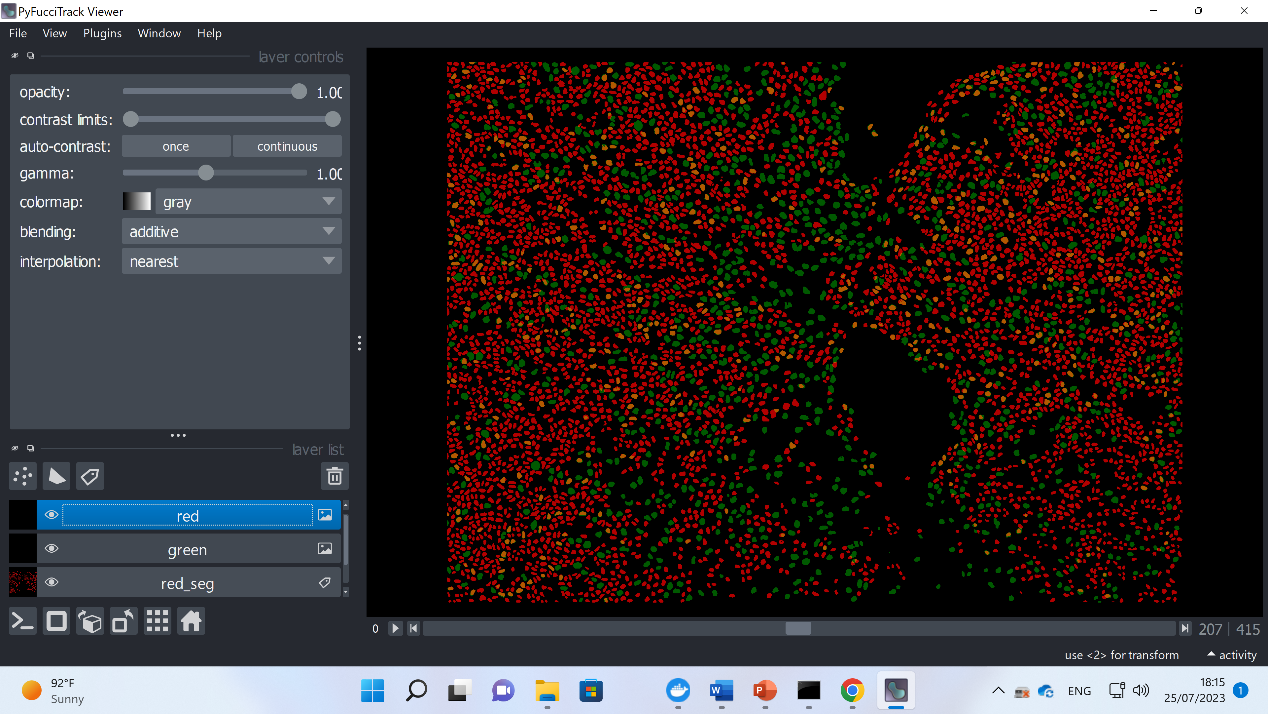

Supplement: S1 File — (DOCX) [file pone.0305491.s001.docx]
